# Infodemic Management Using Digital Information and Knowledge Cocreation to Address COVID-19 Vaccine Hesitancy: Case Study From Ghana

**DOI:** 10.2196/37134

**Published:** 2022-07-12

**Authors:** Anna-Leena Lohiniva, Anastasiya Nurzhynska, Al-hassan Hudi, Bridget Anim, Da Costa Aboagye

**Affiliations:** 1 UNICEF Ghana Country Office Accra Ghana; 2 Health Promotion Division Ghana Health Services Accra Ghana

**Keywords:** COVID-19, infodemic management, misinformation, disinformation, social listening, pandemic preparedness, infodemiology, social media, Ghana, vaccination, qualitative methods

## Abstract

**Background:**

Infodemic management is an integral part of pandemic management. Ghana Health Services (GHS) together with the UNICEF (United Nations International Children's Emergency Fund) Country Office have developed a systematic process that effectively identifies, analyzes, and responds to COVID-19 and vaccine-related misinformation in Ghana.

**Objective:**

This paper describes an infodemic management system workflow based on digital data collection, qualitative methodology, and human-centered systems to support the COVID-19 vaccine rollout in Ghana with examples of system implementation.

**Methods:**

The infodemic management system was developed by the Health Promotion Division of the GHS and the UNICEF Country Office. It uses Talkwalker, a social listening software platform, to collect misinformation on the web. The methodology relies on qualitative data analysis and interpretation as well as knowledge cocreation to verify the findings.

**Results:**

A multi-sectoral National Misinformation Task Force was established to implement and oversee the misinformation management system. Two members of the task force were responsible for carrying out the analysis. They used Talkwalker to find posts that include the keywords related to COVID-19 vaccine–related discussions. They then assessed the significance of the posts on the basis of the engagement rate and potential reach of the posts, negative sentiments, and contextual factors. The process continues by identifying misinformation within the posts, rating the risk of identified misinformation posts, and developing proposed responses to address them. The results of the analysis are shared weekly with the Misinformation Task Force for their review and verification to ensure that the risk assessment and responses are feasible, practical, and acceptable in the context of Ghana.

**Conclusions:**

The paper describes an infodemic management system workflow in Ghana based on qualitative data synthesis that can be used to manage real-time infodemic responses.

## Introduction

The COVID-19 pandemic has led to an unprecedented global “infodemic,” which refers to an abundance of rapidly spreading fake news, misinformation, disinformation, and conspiracy theories related to the pandemic. In the ever-expanding digital world, the infodemic has become increasingly problematic as misinformation spreads rapidly through social media channels [1]. A number of recent studies highlight the negative effects of the infodemic on public perceptions of the COVID-19 pandemic [2-4] and reluctance to comply with public health guidance, including willingness to accept a COVID-19 vaccine [5-7].

Infodemic management has been acknowledged by many public health organizations as an important emerging scientific field and critical area of practice during epidemics [8]. It includes the systematic use of risk- and evidence-based analysis and approaches to manage the abundance of information and mitigate misinformation to reduce its impact on health behaviors during health emergencies. The World Health Organization (WHO) has identified a framework to manage infodemics, which includes listening to community concerns and questions, delivering high-quality health information and programming, building resilience to misinformation, and engaging and empowering communities to take positive action [9]. The WHO is encouraging countries to study and pilot strategies to combat the infodemic surge. As the nature of an infodemic is specific to place and time, it is important to establish a process that identifies context-specific solutions [9].

A growing body of literature on social media platforms has been used to address the infodemic [10-14]. Social media data derived from Facebook, Instagram, Twitter, YouTube, blogs, news sites, and messaging platforms provides useful information to pinpoint context-specific issues in real time to allow for the quick identification of public attitudes on issues of public health importance [10-14]. Gathering social media posts on the basis of a set of keywords, used by digital platforms such as Talkwalker, have become popular with organizations as a means to identify relevant misinformation and rumors [14]. Talkwalker is a dashboard tool that collects, processes, and categorizes information around keywords from social media handles. The UNICEF (United Nations International Children's Emergency Fund) is using this platform to identify misinformation and rumors in several countries [15].

Infodemic management benefits from human-centered approaches that encourage knowledge sharing and knowledge cocreation. While definitions vary widely, knowledge cocreation is essentially the bidirectional, interactive development of new knowledge created with input and perspectives from diverse stakeholders including experts and the public. It allows for the development of acceptable and practical interventions that can be better sustained than those that are developed by public health experts alone [16].

In March 2021, Ghana was the first country worldwide to receive COVID-19 vaccines from the COVAX facility. However, by the beginning of 2022, less than half of the target population of 20 million people had received at least one vaccine dose and only about 13% were fully vaccinated. To increase vaccination rates, the GHS instituted a national COVID-19 vaccination day in February 2022 and inaugurated a second campaign coinciding with Africa immunization week in March of the same year [17]. Surveys during the pandemic indicate that the hesitancy is fueled by different factors that are changing over time, such as the fear of side effects and the lack of trust in the vaccines [18,19]. Similar to many other countries, Ghana has witnessed the widespread transmission of misinformation during the pandemic on the web and offline, including the period during promotion of COVID-19 vaccines [20]. For example, early in the pandemic, COVID-19 misinformation included myths that Black people had some immunity against COVID-19, that the hot climate in Africa reduced the replication of the virus, that COVID-19 was only life-threatening in older people, that drinking “akpetashi”—a locally prepared alcoholic drink—cures COVID-19, and that COVID-19 was a biological weapon to target developed economies; all of which had the potential to reduce risk perception among Ghanaians and contribute to lack of compliance with pandemic measures. There have also been various COVID-19 conspiracy theories identified across Africa on various social media platforms, including those in Ghana, ranging from SARS-CoV-2 having been created as a biological weapon to disrupt the economic power of China against other economically prosperous nations including the United States, to the use of local herbs or products being able to cure the disease [21]. In addition, misinformation has fueled mistrust toward the government, particularly in closed social media platforms such as WhatsApp, which has made risk communication efforts challenging for health authorities during the pandemic [22,23].

Social listening to web-based sources is important in Ghana as the number of social media users has increased significantly in recent years. Currently, over 50% of the population has access to the internet and 140% of the population has a mobile phone connection. In early 2022, there were approximately 8.80 million social media users in Ghana, which is approximately 27.4% of the total 32 million total population. WhatsApp is used by almost 90% and Facebook by over 70% of social media users followed by Instagram by approximately 60% of the users and Twitter and Snapchat are used by approximately 45% of the users [24]. Twitter is known to be used by those who want to generate political discussion in Ghana. The African media agency reports that almost as many women as men use the internet in Ghana, and men are 6% more likely to have a presence on the web than women [25]. However, there are likely to be disparities in the usage of social media between urban and rural populations in the country [24].

The Health Promotion Division of Ghana Health Services (GHS) together with the UNICEF Country Office has established an infodemic management system that combines information identification through the Talkwalker platform and knowledge cocreation among a National Misinformation Taskforce to verify potential misinformation and respond to it appropriately. The system was created to strengthen COVID-19 vaccine programming and to combat vaccine hesitancy, which is defined by WHO as a “delay in acceptance or refusal of vaccines despite availability of vaccination services” [26]. This paper describes the methodology of the infodemic management system in Ghana, which combines digital and human-centered approaches. Some concrete examples are also given to demonstrate how infodemic management operates. The findings of the study can be used to apply the system in other countries that plan to conduct social listening.

## Methods

The infodemic management system was developed by the Health Promotion Division of the GHS and UNICEF Ghana Country Office to effectively identify, analyze, and cocreate content to respond to misinformation during the pandemic. The objectives were to improve compliance with public health safety measures, support COVID-19 vaccine programming, and identify factors that may increase vaccine hesitancy and lead to vaccine refusal.

The data collection system was based on Talkwalker, a commercial social listening software platform. It uses machine learning and artificial intelligence to consolidate publicly visible occurrences of given keywords on the internet. Talkwalker functions like a search engine and provides the ability to filter, contextualize, export, and analyze large data sets. It gathers Ghana-specific COVID-19–related posts from open Twitter, YouTube, and other websites by monitoring keywords, phrases, and hashtags. It categorizes relevant posts by sentiment: neural, positive, or negative. Negative posts are of particular interest as they may contain rumors, misinformation, or disinformation. In addition, the platform includes a feature to categorize data as misinformation; another point of interest to infodemic management. It deepens the understanding of the circulating narratives by aggregating numbers related to the total reach, engagement, and demographic information about those who are engaged in these discussions. The limitation of the tool is that it cannot access conversations on Facebook, Instagram, and WhatsApp owing to privacy restrictions. Approximately 70% of the posts retrieved by Talkwalker in relation to COVID-19–specific information in Ghana are published by men, almost half of which were published by adults aged 25-34 years.

The analysis utilizes qualitative methods to classify a post as misinformation and to assess the risk level of the post. It uses Talkwalker algorithms to identify posts, but the final assessment is based on assessing the post given the local context. In particular, the risk level of particular misinformation requires a qualitative assessment of the situation using applied content analysis [27].

The methodology also relies on knowledge cocreation, which is referred to as collaborative knowledge generation by various stakeholders. Knowledge cocreation is a participatory approach to enhance the value and reliability of outcomes and ensure that they benefit all parties [28,29]. The Misinformation Management Task Force cocreates by assessing the risk level and the proposed responses to address misinformation to ensure they are feasible, practical, and acceptable in the context of Ghana.

## Results

### Working Modalities of the Infodemic Management System

A multi-sectoral National Misinformation Task Force was established to implement and oversee the process developed by the GHS, which also appointed members for the task force. The task force was established on the basis of the membership of an existing task force of Risk Communication and Social Mobilization experts and expanded to include other public health experts, media, development partners, and an organization of Ghana fact-checkers, UNICEF, and other critical partners. Since the beginning of the pandemic, the task force has held biweekly web-based meetings with approximately 20 experts. The head of the Health Promotion Division of GHS is the chairperson of the group. UNICEF has provided technical assistance to the group, including capacity-building training on how to use Talkwalker to identify misinformation, how to assess the risk level of misinformation, and how to respond to misinformation.

**Figure 1:**
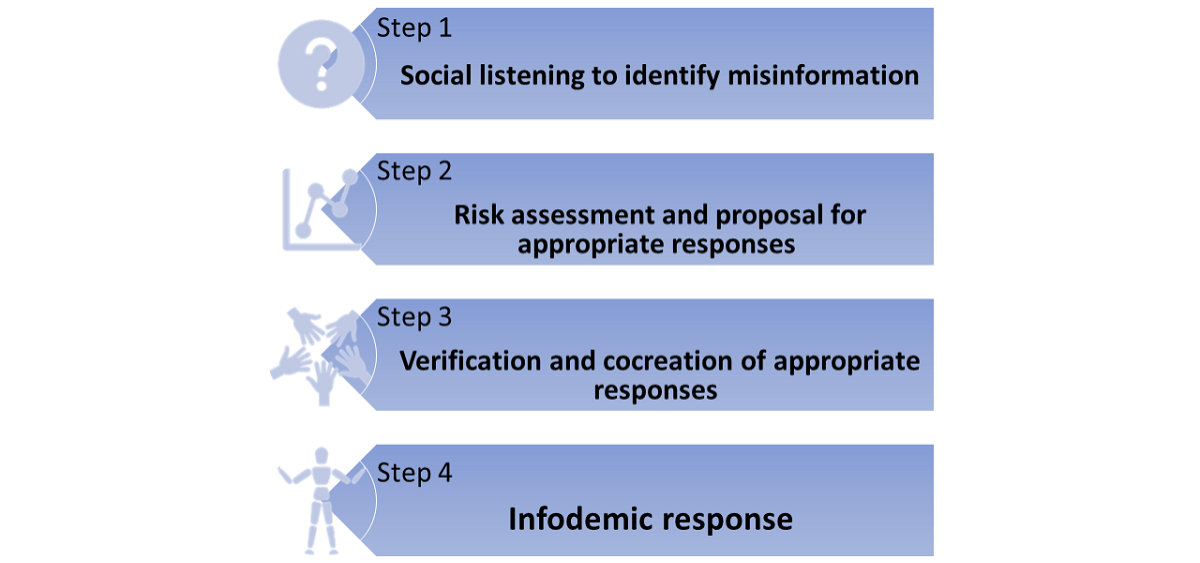
Infodemic management system.

The infodemic management system includes 4 interlinked steps that are carried out by selected members of the task force on a biweekly basis, including social listening to identify misinformation, risk assessment, and proposal for appropriate information; verification; cocreation of appropriate responses; and infodemic response. Figure 1 shows the workflow of the infodemic management system.

### Step 1: Social Listening to Identify Misinformation

The first step is to identify and analyze misinformation through the Talkwalker social media and web-based monitoring platform. Two members of the task force are responsible for carrying out the analysis. They used Talkwalker to determine the number of results (posts that include the keywords for COVID-19 vaccine–related discussions by Talkwalker) during a specific period of time, which is usually a week. The analysts gained an overall understanding of the results by looking into the demographics of those who have generated the results (gender and age) and creating a word cloud to see how the results are thematized. Then, the analysts assessed the engagement rate and potential reach of the results, followed by a review of the results that convey negative sentiments to determine the significance of each individual result. The analyst read each headline of the posts (results) or the entire tweet to decide if it contains misinformation to be included in the analysis. If so, the entire post is extracted from Talkwalker and pasted into a document for further risk analysis and response. Then, the analysts reviewed the rest of the results because even if the reach or the engagement is not high, a result may be potentially risky in the context of Ghana. For example, a post may relate to a historical or political event that is significant in the context of Ghana. At the end of the analysis, the analysts had a list of posts extracted from Talkwalker, which requires verification from public health experts and fact-checkers. If confirmed as misinformation, they are included in the list of misinformation.

### Step 2: Risk Assessment and Proposal for Appropriate Responses

The second step includes assessing the risk level of all the posts that were classified as misinformation based on the UNICEF risk assessment matrix that classifies misinformation into low, medium, or high risk levels based on 5 criteria [30]. See Table 1 for the UNICEF risk assessment matrix. If the analysts are able to link a post to more than 2 criteria in a particular risk level, it is categorized as such. If the analysts are able to relate a post to several levels, the post is classified on the basis of the expert opinion of the analyst based on their broad qualitative analysis of contextual and cultural factors surrounding the post. Once the post has a defined risk level, a set of responses are proposed on the basis of common risk communication and misinformation management best practices [29,31-34].

**Table 1:** UNICEF (United Nations International Children's Emergency Fund) misinformation management matrix (adapted from UNICEF's Vaccine Misinformation Management Guide [30], which is published under Creative Commons Attribution 4.0 International License [35]).

Indicator	Low risk	Medium risk	High risk
Risk to vaccine hesitancy and demand	Low risk to vaccine demand	Potential to trigger vaccine hesitancy	Potential to lead to vaccine refusal
Reach and scope of misinformation	Limited potential to reach scope	Moderate potential to reach scope	Wide cross-country reach or scope
Likelihood of issue spread or escalation	Unlikely to spread on the web or in the community	Spreading on the web or in communities	Spreading rapidly on the web and in community
Capacity to respond	Strong messaging and capacity in place	Some existing messaging and resources to manage crisis	Limited existing messaging and resources to manage crisis
General public trust	Remaining trust in government health services and vaccines	Reduces trust in government health services and vaccines	Increasing mistrust toward government health services and vaccines
Response	Monitor closely and consider prebunking	Debunk and raise trusted voices	Debunk and raise trusted voices

### Step 3: Verification and Cocreation of Appropriate Responses

The analysts presented the analysis in a PowerPoint presentation during the task force meetings. The presentation is discussed jointly with the original post, analysis of the risk level, and proposed response. The discussions are the core of the knowledge cocreation during which the task force members view the contents of the posts and the related risk assessment and proposed interventions and actions to ensure that they are appropriate, culturally acceptable, and practical in the context of Ghana [36]. Decisions are based on consensus among the members of the task force [37].

### Step 4: Infodemic Response

The evidence and the systematic process of verification, risk assessment, and response proposal are provided to the management for approval. There is also a Message Box containing prepared responses to frequently asked questions including rumors, misinformation, and disinformation. Responses may include press releases, social media posts, and direct communication, among others. The GHS is responsible for implementing the response.

### Examples of Implementing the Misinformation Management Workflow

#### Example 1: Negative Attitude of Health Care Workers

Through Talkwalker, the analysts identified a post that included complaints about negative attitudes of health care staff toward patients in one particular health center. The owner of the post was a young social media influencer with over 10,000 followers, many of whom also actively retweeted the post within their own networks. Analysts considered that a risk, though the allegations of the post itself were not considered particularly threatening as it related to one particular health center. The analysts suggested taking localized action to address the issues with that particular health center. During Misinformation Task Force cocreation, the members rated the post as medium risk in consensus and agreed to take targeted action by training all staff members of that particular health care center in service delivery and customer care.

#### Example 2: Misinformation About the Side Effects of COVID-19 Vaccines

Through Talkwalker, analysts identified a post on popular news sites about an interview with a local premier league football team coach in which he claimed that the team had lost a game because of their weak physical status after they received the COVID-19 vaccine. Analysts noted that the engagement rates were high and this spread rapidly on social media platforms, particularly on Twitter. The analysts rated it as high risk because it was from a national web-based news outlet, because the interview was with a local celebrity, and because football is a popular sport in Ghana and many fans may be influenced by the post. The analysts suggested taking action on the web in the same news outlet where the post was published. The task force agreed with the high risk level but instead of responding on the web, they decided to contact the football coach directly to gain clarification on his statement, to educate him about the side effects of the COVID-19 vaccine, the Adverse Effects Following Immunization protocol, and, most importantly, to recruit him as a vaccine supporter and encourage him to speak publicly to deliver pro–COVID-19 vaccine messages.

#### Example 3: Disinformation About the Alleged Lethal Nature of the COVID-19 Vaccine

Using Talkwalker, the analysts identified a retweet about a post that claimed to be published by an award-winning doctor who warned that those who take the COVID-19 vaccine will die within 2 years. The analysts checked with Ghana fact-checkers about the post and learned that it had been viral worldwide for some time already and it had been fact-checked as false. The analysis rated the post as high risk owing to the global spread and because it referenced death. They proposed to the task force that action should be taken to clarify that this was fake news. The task force agreed that the post should be rated as high risk because it described a severe adverse effect, which is known to promote vaccine hesitancy, and because the disinformation claims to have originated by a doctor—a highly respected profession in Ghana—which could contribute to the spread of this misinformation in Ghana. The task force decided to circulate the post with a “Fake News” stamp across various GHS social media channels. At the same time, they posted factual information about the COVID-19 vaccine and had it circulated across social media channels.

#### Example 4: Mistrust Toward the COVID-19 Vaccination Program and Health Authorities

Through Talkwalker, the analysts identified a Tweet that accused the GHS of not sharing information about COVID-19–related mortality on its website in a timely manner. It was created by a political activist, with over 20,000 followers, who is known to initiate discussion against the government. The analysts assessed the risk as medium as they did not find any tangible accusations in the post and suggested that no action be taken at this time as a response would only bring more attention to the post. The task force assessed the risk level as high because the post could encourage more politically driven rumors to further spread mistrust against the government. The task force response included issuing an official press release that clarified the data verification process of any statistics displayed by the GHS on its website and highlighting the importance of publishing accurate information. A summary of the examples is provided in Table 2.

**Table 2:** Examples of infodemic management systems in Ghana.

Talkwalker	Risk assessment	Action	Expected outcome
The attitude of nursing staff in xx health unit is not appropriate. People are not willing to get their COVID-19 vaccines at that location.	Medium risk: potential to increase COVID-19 vaccine hesitancy as people are unwilling to get the vaccine in the health unit. It also impacts the uptake of any services at that particular health unit.	Training of the health care unit staff members in customer service.	Improved health systems through more service-oriented staff.
Misinformation was spread by a national football coach who claimed having lost a game because all players were vaccinated and it made them weak.	High risk: football is a popular sport in Ghana and the coach is seen as a local celebrity. Accordingly, the misinformation can spread rapidly among football fans.	Personal contact with the football coach to understand his claims, provide information about the COVID-19 vaccine, and encourage him to publicly advocate for the vaccine.	Gaining the football club as a vaccine supporter that can disseminate positive COVID-19 vaccine messages as needed.
Disinformation by an alleged doctor that all who have taken the COVID-19 vaccine will die in 2 years.	High risk: potential to increase vaccine hesitancy and contribute to refusal to take the vaccine because it was from an alleged doctor and relates to the severe adverse effect of the vaccine. The disinformation was also circulating widely and rapidly.	Fact-check and, once verified fake, circulate the news with a fake news stamp. Simultaneously run factual information about the COVID-19 vaccine across different social media platforms.	Stopped circulation of the fake news.
Rumors that GHS is faking COVID-19 death statistics as the numbers on the website do not correspond with numbers available on social media.	High risk: potential to decrease trust toward the COVID-19 vaccination program and Ghana health services.	Issue a press release and explain that sometimes there is a lag in GHS numbers owing to the verification process to ensure that the numbers are correct and highlight how important it is for GHD to verify any information before publishing it on the website.	Improved trust towards GHS reporting procedures.

## Discussion

### Principal Findings

This paper described an infodemic management system developed and implemented in Ghana. The system relies on data collection through a digital platform and on human-centered approaches to verify the findings with appropriate response mechanisms. The system has been used to identify COVID-19 misinformation, disinformation, and rumors, which were addressed in a timely manner.

The implementation of the infodemic management system in Ghana highlights the critical role of qualitative inquiry in social listening as it allows for a greater understanding of the positions, perceptions, and potential misinformation and disinformation among population groups in order to assess the potential risk and take appropriate action in a timely and targeted manner. For example, a post from a football coach may not be significant in a country where football is less popular, but in the context of Ghana, it was assessed as a high risk that has the potential to spread fast and raise high emotions. Talkwalker cannot carry out such an interpretation, which aligns with a number of studies that have pointed out the limitations of machines. Although machine learning methods have been developed to solve real-world problems, they are not sufficient by themselves in critical decision-making approaches [38,39]. Digital platforms still have limitations to interpret and contextualize data [40]. In addition, digital platforms cannot commonly identify whether the information is misinformation or disinformation; a critical differentiation essential to infodemic response processes [29]. The use of a digital platform together with a qualitative analysis aligns with a UNICEF MENA (UNICEF in the Middle East and North Africa region) case study that showed the importance of involving human minds in digital data interpretation to create a shared sense of reality that fosters engagement and connections with the communities and facilitates risk communication and community engagement responses [15].

The implementation of the infodemic management system in Ghana has also highlighted that knowledge cocreation can be implemented even in crisis situations. Knowledge cocreation has been identified in a number of studies as an effective approach to discover, share, and blend knowledge for practical use, allowing stakeholders to learn about the applied implications of knowledge use and to collectively create actionable recommendations [41,42]. Cocreation can act as capacity-building for those who participate [43]. Ideally, cocreation will allow the task force members to build their misinformation management skills so that in the future, the system can run without support from external stakeholders such as UNICEF. A systematic approach to detecting, analyzing, and responding to an infodemic also often facilitates official approvals for press releases or other responses [12]. The Misinformation Task Force was developed by merging an existing working group with the task force instead of creating a new structure, which has been a successful model in other countries such as Finland where social listening was built into existing working groups [12].

The infodemic management system in Ghana also has limitations. The Talkwalker posts and interactions are mainly published by men and young adults, excluding the voices of women, youth, and older people. In addition, here are still significant numbers of people, particularly in vulnerable populations such as low-income individuals and those who cannot read and write, who are not reached by digital platforms [44]. Accordingly, there is a need to merge offline listening systems with the infodemic management system, such as the perspectives of community leaders, who are highly respected in Ghana, and women, who play a key role in the vaccination decision-making of their children in Ghana [45,46]. Moreover, Talkwalker does not include Facebook or WhatsApp, which are two of the most popular social media sites in Ghana [21]. The qualitative inquiry of the system has also weaknesses. The process of identifying misinformation relies on the analyst’s decision and is based on their own reflectivity including their worldview, beliefs, attitudes, and skills [47], which may present bias as to what type of information is determined as disinformation or misinformation and how significant risk it is perceived. Cocreation methods can mitigate bias [48]. Other strategies to minimize bias should be considered, such as engaging more task force members with different backgrounds in the assessment and analysis process [48]. In the future, studies should be conducted to measure the impact of the system and the various infodemic response strategies implemented by the task force.

### Conclusions

The paper has described an infodemic management system workflow based on a mix of digital and human-centered methods, including effective social listening through a social media management platform, qualitative analysis process, and cocreation through a national task force of experts, resulting in context-specific, real-time infodemic responses.

## Abbreviations

GHS: Ghana Health Services
UNICEF: The United Nations International Children's Emergency Fund
UNICEF MENA: The United Nations International Children's Emergency Fund in the Middle East and North Africa region
WHO: World Health Organization
